# Morphological and chemical characterization of nanoplastics in human tissue

**DOI:** 10.21203/rs.3.rs-6166886/v1

**Published:** 2025-03-12

**Authors:** Aaron Erdely, Vamsi Kodali, Marcus A. Garcia, Kristin Bunker, Long Li, Jim Marquis, Alan Levine, Michael Deible, Tracy Eye, Alexander J. Nihart, Natalie L. Adolphi, Daniel F. Gallego, Eliane El Hayek, Matthew J. Campen

**Affiliations:** 1 Health Effects Laboratory Division, National Institute for Occupational Safety and Health, Morgantown, WV, USA; 2 Department of Pharmaceutical Sciences, College of Pharmacy, University of New Mexico Health Science, Albuquerque, NM, USA; 3 RJ Lee Group, Pittsburgh, PA, USA; 4 Office of the Medical Investigator, University of New Mexico, Albuquerque, NM, USA

**Keywords:** microplastics, brain, kidney, liver, dimensions, bioaccumulation

## Abstract

Micro- (≤ 5 mm) and nano- (≤ 1 μm) plastics have become ubiquitous resulting in inevitable human exposure. Evidence exists of mass-based accumulation of plastic in human tissues with visualization of micron-sized particles (> 1 μm). To date, there is little evidence to address accumulated nanoplastics. Understanding internalized plastic particle morphological and chemical characteristics is essential to facilitate proper design of future mechanistic and controlled exposure health effects studies to determine whether any health-related risks exist. Here we show microscopic evidence and quantitative dimensional analysis of nanoplastics in human decedent brain, kidney, and liver tissues. Mean particle lengths (nm) across the five decedents were 171.2±4.6 for brain, 124.4±3.6 for kidney, and 147.6±6.6 for liver. Mean particle widths (nm) were 45.9±1.5 for brain, 32.3±0.7 for kidney, and 36.1±1.3 for liver. When examining the aspect ratio, 78–83% consisted mostly of an elongated nanometer sized fiber morphology. The study provides isolation with physical and chemical characterization of nanoplastics in human tissues. Interestingly, differences were greater between tissues of a single decedent than across decedents. Consistently, the nanoplastics were largest in the brain. The observations overall suggest specificity with respect to systemic internalization and subsequent tissue accumulation of plastic particles less than one micron.

## Main

Globally, more than 450 million metric tons of plastic are produced annually^1^. While now critical to daily life in most countries, concern is growing as to the fate and consequences of plastic waste, especially as it degrades into micro- and nanoplastics (MNP). Microplastics are defined as particles with a size ≤ 5 mm and nanoplastics ≤ 1 μm in length^2^. Due to ever increasing use, MNP concentrations are rising in the environment, with a doubling time of approximately 10–15 years^3,4^ and also potentially increasing in the human body^5^.

There is currently limited clarity on whether human MNP exposure would result in adverse health effects. Numerous studies have confirmed the presence of MNP in human fluid/waste including blood, semen, breastmilk, feces, and urine^6–12^. More recently, MNP have been confirmed in internal tissues including testis, blood vessels, bone marrow, lung, olfactory bulb, synovium, placenta, liver, stomach, brain, tumors, and cardiac tissue^5,11,13–26^. MNP presence in the blood and carotid or coronary atherosclerotic plaques was associated with inflammation and increased cardiovascular risk suggesting potential correlation to adverse health outcomes^12,22,27^.

Initial studies relied on tissue concentrations of plastic with limited microscopic observations. Virtually all studies referenced above for human sampling used methods (e.g., filter pore size > 1 μm) or visualization techniques that were selective for identification of microplastics, meaning the particle needed to be greater than a few microns to confirm with Raman or Fourier transform infrared spectroscopy (FTIR) spectroscopy. Controlled *in vivo* studies suggest that nano-sized plastics would be more likely than micron-sized to be distributed to internal organ systems following exposure^2,28,29^. Thus, while initial studies confirmed the presence of MNP throughout the body, the technical limitations of contemporary spectroscopy (e.g., Raman, FTIR) meant that there was limited information on the presence of nanoplastics.

The primary focus of this study was to systematically evaluate the nanoplastic fraction that was present in various selected human tissues. We hypothesized that accumulation would vary between tissues dependent on physical dimensions of the particle with the brain being the smallest due to the highly selective blood brain barrier. It is imperative to understand the morphology of the plastic particles that are present in human tissues to inform pathways of uptake, accumulation, and clearance, and whether nanoplastics will initiate or exacerbate any human health issues. Characterization is also essential to facilitate proper design of future mechanistic and controlled exposure health effects studies to determine whether any health-related risks exist^2,30–32^.

## Isolation reveals nanoscale particulates

**Figure 1:**
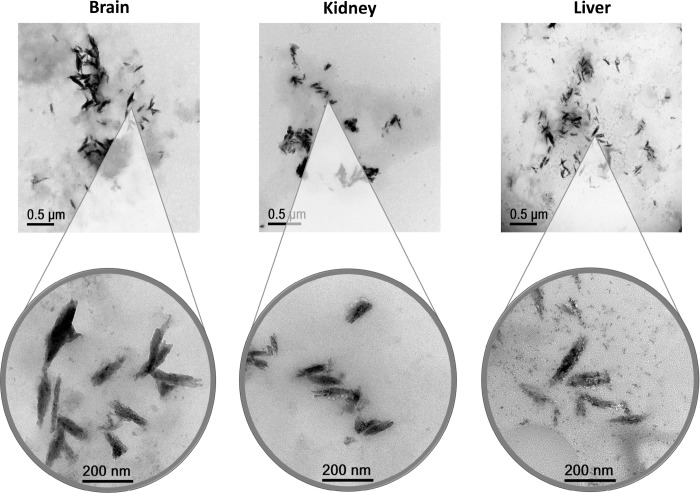
Representative high resolution transmission electron microscopy imaging of nanoparticulates isolated from human brain (frontal cortex), kidney (piece containing cortex and medulla), and liver (right central parenchyma).

Details on the selection of decedents, the sampling methods, and the number of specimens assessed can be found in Nihart et al., 2025^5^. The n=5 selected decedents were those with all three tissues available. Collected tissue included the brain, liver and kidney. The human brain, liver, and kidney samples evaluated in this study, n=5 of each tissue from five decedents (Table S1), were known to contain polymer solids as determined by pyrolysis gas chromatography mass spectrometry (Py-GC-MS)^5^. The total average polymer mass concentration of the samples used ranged from 70–5731 μg/g, with brain concentrations being greater than either kidney or liver, and the speciation was predominately polyethylene, polypropylene, and polyvinyl chloride (Figure S1). Using the isolation and resuspension protocols described in the Methods, visualization of the nanoparticulates was performed using transmission electron microscopy (TEM). Clear evidence of irregular shaped particles was observed in the different tissues (Figure 1, Figure S2–4), consistent with observations recently published^5^.

## Confirmation of polymer nature

**Figure 2:**
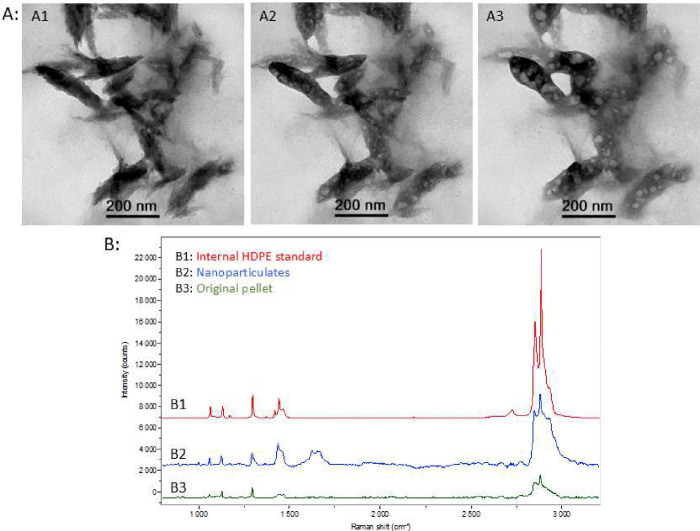
**A**: To confirm their polymer nature, nanoparticulates from the brain were imaged and mapped to enable relocation after exposure to the solvent vapors and heating. The grid was exposed to chloroform vapors for 24 hours as described in Supplemental Figure 7. Following exposure to the chloroform vapors, the grid was placed into an oven at 130°C for 24 hours. The particles were imaged before exposure to chloroform (A1), after 24 hours of exposure to chloroform (A2), and after heating at 130°C for 24 hours (A3). **B:** A sample for Raman spectroscopy was prepared as described in the methods by using particles from liver dispersed in equal volumes of isopropanol and benzene. Spectra collected included internal high-density polyethylene (HDPE) standard (B1, red), particle sample using 600 second exposure with 10 accumulations and 25 μm hole (B2, blue), and the original isolated pellet sample using 180 second exposure with 3 accumulations and 25 μm hole (B3, green).

Py-GC-MS suggested that the tissue samples contained various polymer types of differing mass concentration (Figure S1). To more confidently conclude that the observed nanoparticulates were polymer, further confirmation tests were performed (Figure 2; Figure S5–10). First, the nano-sized particles were predominantly carbon and oxygen in nature compared to areas lacking particles using energy-dispersive X-ray spectroscopy (EDS) (Figure S5). Salts, as expected, were also evident in the preparation likely originating from the biological material, residual additives from plastic production, digestion procedure, as well as the 10% neutral buffered formalin. As a test only, suspension in isopropanol and filtration using a 100 nm filter solubilized and reduced the salts, while retaining the particles on the filter that did not dissolve in isopropanol (evidenced in TEM images and filtrate salt measured by inductively coupled plasma analysis; data not shown).

After dispersion in isopropanol, aliquots of samples were added to an equal volume of benzene. Benzene was chosen as a solvent due to the lack of compatibility with polyethylene, the largest fraction of plastic present in the samples, as well as other plastic types. Benzene clearly influenced the structure and morphology of the particles when directly combined (Figure S6). Understanding that organic solvents would affect the particles with time, vapor dissolution experiments were performed using electron microscopy with mapping to allow for relocation before and after. Both chloroform and benzene had dissolution effects on the nanoparticulates that were measured serially up to 471 hours of vapor exposure (Figure S7). Heat (130°C) was added to the TEM grid used for the chloroform vapor exposure test (Figure 2A). The heating clearly showed swelling and fusion of the nanoparticulates with loss of individual particle characteristics which became larger particles appearing to join together.

The isolated and dispersed particles, washed subsequently in benzene and heated to further remove any potential contaminating biological materials, had a spectral profile largely mirroring polyethylene, the most abundant plastic accumulating in the decedent tissues (Figure S1), albeit with a peak from 1600–1700 cm^−1^ consistent with elevated carbonyl content when examined by Raman spectroscopy (Figures 2B and S8). The particles also had a similar profile consistent with polyethylene using Fourier-transform infrared spectroscopy (Figure S9).

The use of an organic solvent also reduced the potential of lipid contamination. Cholesterol crystals, generated in the laboratory to rule out an obvious potential biological artifact, were readily dissolved by benzene as expected (data not shown). This observation illustrates a direct contrast to the very slow dissolution of the nanoparticles derived from tissues which still had integrity up to one week in benzene solution (Figure S6) and 20 days (471 hours) in the vapor dissolution test (Figure S7).

Alternative methods not using potassium hydroxide can be used to process samples. A brain sample was digested in hydrogen peroxide and nitric acid, ultracentrifuged, and visualized using transmission electron microscopy. Particles with similar dimensions were observed (Figure S10). Overall, our chemical characterization supported the notion the observed particles were indeed polymer in nature.

## Different particle size across tissues

**Figure 3:**
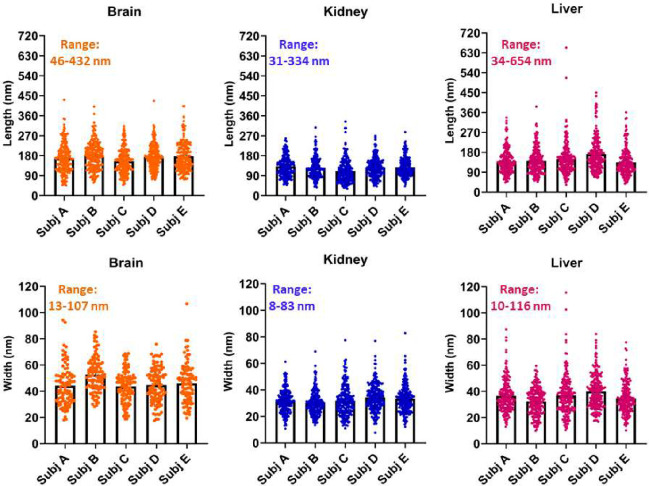
Physical dimensional profiling of nanoparticulates isolated from human brain, kidney, and liver. A total of 3251 measurements were made with n=205–231 individual measurements per decedent per tissue sample. Samples used for particle size quantification were prepared using isopropanol, which does not affect the polymer fragment dimensions, without filtration.

When considering all measured particles from all sampled tissues, the range for the length and width, respectively, were 46–432 nm and 13–107 nm for brain (n=1103), 31–334 nm and 8–83 nm for kidney (n=1075), and 34–654 nm and 10–116 nm for liver (n=1073) (Figure 3). Our analysis of 3251 different singlet particles from these 3 organs from 5 decedents indicated all dimensions to be less than 1 μm. Size variability between decedent subjects was modest, while the clearer observation was the nano-sized particles in the brain were consistently longer and wider than the kidney or liver.

**Figure 4:**
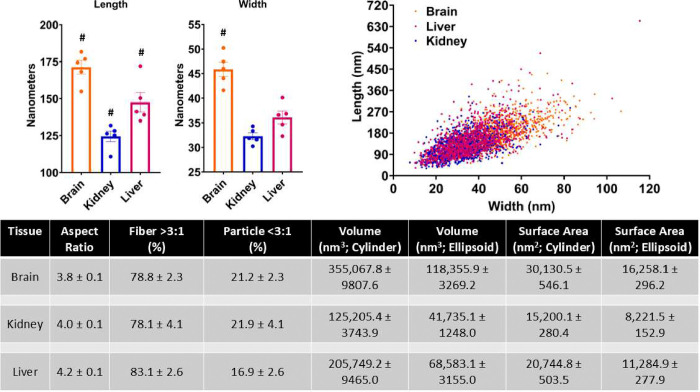
(**A**) Average length and width for nanoparticulates isolated from human brain, kidney, and liver (n=5; #p<0.02 vs all groups). (**B**) Size-distribution pattern of isolated particles illustrating longer length and width associated with brain accumulation. (**C**) Table illustrating aspect ratio and percentage of particles meeting the fiber criteria. Additional calculations offer estimated average volume and surface area occupied by a particle.

The mean ranges for particle length and width, respectively, of the five decedents were 154.9–181.7 nm and 41.6–50.2 nm for brain, 110.8–131.8 nm and 30.3–34.3 nm for kidney, and 135.0–172.0 nm and 32.3–40.1 nm for liver. The means of the five separate decedent for particle length were 171.2 ± 4.6 nm for brain, 124.4 ± 3.6 nm for kidney, and 147.6 ± 6.6 nm for liver (Figure 4A; #p<0.02 different from all groups). The means of the five separate decedents for particle width were 45.9 ± 1.5 nm for brain, 32.3 ± 0.7 nm for kidney, and 36.1 ± 1.3 nm for liver (Figure 4A; #p<0.001 different from all groups). The distribution profile of the paired length and width measurements clearly indicate the brain contains on average larger particles (Figure 4B). The liver has some larger particles, but comparatively these were more the exception than the norm. When examining the aspect ratio (Figure 4C), the particles, 78–83%, consisted of fiber morphology (greater than a 3:1 aspect ratio) as opposed to irregular particle shapes (aspect ratio less than 3:1; 17–22%). Estimated volume and surface area calculations, assuming a cylinder or ellipsoid (thickness assumed to be one half the width), reflected greater availability for particle contact with brain tissue.

Size distribution histograms indicated generally normal distributions of physical dimensions with similarities between decedents despite varying demographics (Figure S11A; Table S1). The kidney had the narrowest overall distribution while the brain had the largest. Due to the general similarity of physical dimensions between decedents, all samples were considered together (Figure S11B). With more than a thousand individual particle measurements for each tissue type, the particles found in the kidney were skewed to the left (smaller size) while the brain was broader and populated the greatest proportion of the larger size ranges. The particles in the liver were intermediate in size.

## DISCUSSION:

Our study illustrates a consistent nanoscale polymer particle presence in the tissues of the five examined decedents. The results are distinct from studies that observed plastic in human specimens using techniques incapable of visualizing nano-sized particles and/or used filters with collection pore sizes too large (> 1 μm) to capture the nano-sized fraction. The present observation parallels recent findings of environmental nanoplastics in seafood, insect meal, and water^33–35^ and provides further visual and chemical characterization. A previous complementary study revealed irregular jagged particles of foreign origin, presumably plastic, in atherosclerotic plaques using transmission and scanning electron microscopy^27^. The authors of that study qualitatively determined that the size range accumulating was < 200 nm supporting the quantitative results of this study. These small sizes found in the human organs are not much larger than viruses, which complicates our ability to visualize and characterize the material *in situ* but offers insights into potential implications for health.

Controlled *in vivo* rodent studies have shown that systemic plastic uptake inversely relates to particle size following ingestion, with nano-sized plastic surrogate materials accumulating more than micron-sized particles^2,28,29^. Various *in vivo* studies have shown systemic distribution of nano-sized plastic particles following an inhalation or ingestion exposure including the placenta, the brain, and various other systemic organs beyond the site of exposure^2,29,36,37^. To offer insight into exposure pathways for humans, Fraissinet and colleagues, using a six-step filtration process following digestion, found the nano-sized fraction (< 400 nm) to be a substantial fraction of the plastic mass concentration found in mussels^33^, a study reflecting how MNP accumulate in biological tissues. This observation would also suggest a significant particle number to contribute to the mass, as it requires orders of magnitude greater numbers of 100 nm sized particles to equal the mass from a single 5 μm particle. Further, the largest nano-sized plastic mass reported was the 20–200 nm fraction, with polypropylene, polyethylene, and polyvinyl chloride being the most abundant types of plastic, paralleling the human samples analyzed in this study. This observation suggests that the presentation and subsequent accumulation of plastic in human tissues and type can be predetermined by the diet consumed and may already be a highly selected size range due to bioaccumulation up the food chain^38^. Dynamic release modeling from an industrialized country indicate that the same plastics observed mostly in human tissues, including polyethylene, polypropylene, and polyvinyl chloride, were of the highest plastic consumption and stock levels^39^. Cumulative release to environmental sinks since 1950 indicated agricultural soils were the most significant for microplastics, with polyethylene being the largest fraction^39^. Further research to determine the nanoparticulate fraction, especially in agricultural settings, is warranted.

We incorrectly hypothesized that the brain would have the smallest size fraction of accumulated particles given the stringency of the blood brain barrier. This was interestingly not the case as the kidney consistently had the smallest size fraction. In a previous study, human brain samples were shown not to exhibit an increasing concentration relationship with age for plastic, although the date of death did suggest a parallel with increasing environmental concentrations^5^. In complement, a study in zebrafish^40^ suggested that a steady state internal plastic concentration can be achieved, but the plateau concentration in the body was dependent on external exposure concentrations. Additionally, concentrations in zebrafish declined with removal of the plastic exposure. These studies suggest equilibrium of uptake and clearance, not continued accumulation, supporting similar physical dimensions in each decedent tissue despite the wide age range evaluated. Our observations further suggest that uptake, retention, and clearance mechanisms may meaningfully differ between organs and the mechanisms need examined. The liver and kidney are organs designed for clearance and as such may be more effective at removal of larger particulates consistent with the smaller size and less mass based accumulation compared to the brain. Irrespective, the study observations indicate an understudied size fraction of potential concern when considering potential human health outcomes, as nanoscale particulates can reside inside and between cells and even alter cellular function and structure which can be further complicated by environmental and/or biological coronas^41,42^. Future preclinical research may consider the use of appropriately sized and shaped materials to better predict health risks^32,43^. The results clearly define a size fraction with propensity for human accumulation while importantly provide a size range of interest to measure externally, such as in environmental or occupational settings.

## LIMITATION:

The quality control for collection, storage, digestion/isolation, contamination, and measurements, including procedural controls, for the samples analyzed have been detailed^5^. Our extensive confirmation tests failed to exclude the particles as something other than a polymer. Higher beam intensity with transmission electron microscopy was avoided to limit beam damage suggesting plastic, while the EDS indicated significant carbon and oxygen content. Both Raman and FTIR spectra were representative of plastic but cannot completely rule out lipid contamination similar to concerns with Py-GC-MS in biological matrices^44^. The temporal stability of the nanoparticulates in organic solvents compared to potential biological artifacts (e.g., laboratory generated cholesterol crystals) argues that the particles are plastic polymers. We found nanoparticulates with KOH digestion as well as alternative digestions (both H_2_O_2_ and nitric acid), combined with the distinct protocol to isolate the nano-sized plastic particles from mussels that used filtration instead of ultracentrifugation^33^, suggest the particles are not a digestion artifact. Had the particles been a procedural artifact, the expectation would have been dimensional uniformity across all tissues given they were handled and processed similarly. More widespread analyses are warranted beyond the n=5 from this study, but this study offers a critical beginning step. We would suggest a wider geographical range considering the consistency of data between subjects despite the vast age range.

The spectral data of the particles could suggest an advanced aging/degradation of plastics, consistent with the high level of the potential carbonyl peaks seen by Raman spectroscopy. More extensive analysis is needed to confirm the reason for the peak, which is not seen in the reference sample of pristine polymer or the initial tissue pellet. It could be an artifact of organics used during processing, but it may represent an oxidation state of the particles representative of what may be expected from extensively aged plastics. The latter would suggest that the observed nanoparticulates are not derived from fresh plastics, but from decades-old plastics waste, an important consideration for sustainability, as well as well as for analytical methods for detection and quantitation. That would also imply the particles we observed would be more correctly termed fragments. The concept of an increased equilibrium mass of plastic accumulation in human tissue with progressing years^5^ would coincide with increased plastic production with time and be consistent with increased degradation time (aged/weathered) for increased environmental presence of nano-sized plastic.

## CONCLUSION:

Physical dimension profiling of nanoparticulates of polymer composition in human tissue revealed a narrow nanometer size range and insights into the nature of uptake and distribution in the body. Interestingly, and quite remarkably, the accumulated size difference was greater between organs of a single decedent than between decedents. Consistently, the particles were larger in the brain. The observations overall suggest some specificity with respect to systemic internalization, retained tissue fraction, and clearance mechanisms, while offering a size fraction to include for future biomonitoring and research. We used specific methodology to analyze the nano-sized fraction in contrast to previous reports centered on visualization and chemical characterization of micron-sized plastics. The variances in size fractions should be considered in complement, with appreciation the micron-sized particles will be few while the nano-sized fraction constitutes many orders of magnitude more particles by number.

This study does not imply any health impact associated with the accumulated nanoplastic particles. Studies are emerging of potential associations with plastic accumulation and cardiovascular disease ^12,22,27^, neurological disease^5,45^, cancer^26,46^, liver disease^18^, and gastrointestinal disorders^10^. Prior to any causation, understanding the morphological and chemical nature of accumulated plastic in humans is critical. Additionally, research is needed to understand the environmental, occupational, agricultural, and dietary pathways that nanoplastics travel to gain access to the human body.

## METHODS

### Human Samples:

Available brain (frontal cortex), kidney (piece containing cortex and medulla), and liver (right central parenchyma) were obtained from five de-identified decedents retrospectively by the University of New Mexico (UNM) with approval from the Office of the Medical Investigator and UNM Human Research Protection Office (HRPO). This activity was reviewed by the UNM HRPO and deemed research not involving human subjects. Consistent regions from all organs were collected by a forensic pathologist. The frontal cortex was chosen because of the potential link to neurodegenerative diseases. The collected tissues (roughly 3–5 cm^2^) were collected in 10% buffered formalin. Decedents (n=5) were 19–76 in age range and 1 male and 4 female (Table S1). The goal was to utilize samples from randomly selected decedents which had more than one tissue available for evaluation to allow within individual tissue comparisons^5^. Available demographic information was limited due to the conditions of specimen approval (Table S1).

### Isolation:

Tissues (n=15; ~500 mg each) were digested using 10% potassium hydroxide (KOH) at 40°C for at least 3 days. Samples were then ultracentrifuged at 100,000 times gravity, g, for 4 hours. The obtained pellet has been shown to contain materials consistent with polymers as pleasured by Py-GC-MS. Those results indicate specific total mass and speciation of different polymers (Figure S1). The method used to produce the mass and polymer type consumes the sample. To understand the morphology, a separate 500 mg sample was isolated, and the obtained pellet was resuspended and visualized by transmission electron microscopy using a modification of previously established methods for generating singlet carbon nanotubes and carbon nanofibers^47,48^. Pellets (Figure S12) were resuspended in a glass vial with 2 mL of filtered isopropanol. The pellet is a mixture of residual biological material and particles. The sample was sonicated using a water bath sonicator for a duration of 30 minutes followed by a 7-day incubation at room temperature.

### Polymer confirmation tests:

We conducted tests including energy-dispersive X-ray spectroscopy, inductively coupled plasma analysis, organic solution and/or vapor incubations (benzene and chloroform), Raman spectroscopy, Fourier transform infrared spectroscopy, as well as alternate digestion procedures. The detailed methods are contained in the Figure legends of the Supplemental Figures. Of note, filtration for salt determination / dissolution and use of organic solvents were for polymer confirmation testing only. Particle size quantification samples were prepared using isopropanol, which does not affect the particles dimensions suspected to be polymeric in nature, without filtering.

For Raman spectroscopy (Figure 2), a sample was prepared by using nanoparticulates from liver dispersed in equal volumes of isopropanol and benzene. The vial was heated in a heating block at a temperature of approximately 78° C and monitored by a thermocouple. Several drops were deposited onto an aluminum coated slide. Samples were analyzed using a Horiba LabRam HR with an Olympus BX41 microscope with a 50x LWD lens. Analysis was conducted using a 632 nm excitation source and 600 gr/mm grating.

### Measurements:

Filtration was not performed to segregate any collection size ranges prior to size quantification. After 7 days, samples were sonicated again for 30 minutes. Following sonication, samples were placed onto a copper transmission electron microscopy (TEM) grid coated with a thin layer of carbon. Using an optical microscope to determine loading, 1–15 drops (~4 μl / drop) were added to the grid allowing drying between drops. Imaging was performed at 120kV on a JEOL JEM-1400 LaB6 TEM equipped with Gatan Rio CMOS camera for imaging. Imaging revealed nano-sized irregular shaped particles. Physical dimension profiling of the nano-sized particles was done for each tissue for each decedent (n=205–231 individual measurements / tissue randomly selected) with a total of 3251 measurements performed. Length and width were measured for each particle. The longest end to end measurement was measured as the length and the widest portion of the fragment was represented as the width. Aspect ratios were then evaluated with proportions representing fibers, 3:1 or greater aspect ratio, or particles, less than 3:1 aspect ratio, determined.

### Statistics:

One-way analysis of variance (ANOVA) with Tukey’s HSD post-hoc test was performed to evaluate differences between human samples of the same tissue and differences between tissues. Data will be available at https://data.cdc.gov.

## Data Availability

Data will be available at https://data.cdc.gov.
